# Extracranial hypoglossal nerve schwannoma presenting as a submandibular gland tumor: diagnostic pitfalls and surgical considerations—a case report

**DOI:** 10.3389/fsurg.2026.1973163

**Published:** 2026-10-26

**Authors:** Jun Ren, Min Tang, Lijuan Guo, Sen Yang

**Affiliations:** Department of Oral and Maxillofacial Surgery, Suining Central Hospital, Suining, Sichuan, China

**Keywords:** case report, diagnostic pitfall, hypoglossal nerve, pleomorphic adenoma, schwannoma, submandibular region, surgical management

## Abstract

Schwannomas are benign, encapsulated tumors arising from Schwann cells. Extracranial hypoglossal nerve schwannomas located in the submandibular region are exceptionally rare and may clinically and radiologically mimic primary submandibular gland tumors. A 44-year-old woman presented with a painless left submandibular mass of three months' duration. Ultrasonography and contrast-enhanced computed tomography demonstrated a well-circumscribed heterogeneous lesion closely associated with the left submandibular gland, and a pleomorphic adenoma was suspected preoperatively. Preoperative needle biopsy was not performed because of concern regarding sampling error and a potentially nondiagnostic result. Magnetic resonance imaging was not performed because of financial considerations and the patient's reluctance to undergo further examination. During surgery, the encapsulated tumor was found to arise from the hypoglossal nerve and was carefully dissected from the nerve sheath while preserving the anatomical continuity of the main nerve trunk. Histopathological and immunohistochemical examinations confirmed schwannoma. At the one-year follow-up, the patient showed normal tongue movement without recurrence or neurological deficit. A review of published reports indicates that extracranial hypoglossal nerve schwannomas have been reported to present as painless submandibular masses and may be misdiagnosed before surgery. This case emphasizes that preoperative recognition of hypoglossal nerve origin may facilitate appropriate surgical planning and may allow preservation of both the functional nerve and the uninvolved submandibular gland.

## 1 Introduction

Schwannomas are benign, slow-growing, and usually encapsulated tumors derived from Schwann cells (1). Approximately 25%–45% of schwannomas occur in the head and neck region (2); however, extracranial schwannomas originating from the hypoglossal nerve are uncommon. Most hypoglossal nerve schwannomas arise intracranially or near the skull base (3, 4), whereas those presenting as isolated submandibular masses are exceptionally rare.

Because extracranial hypoglossal nerve schwannomas often present as painless, slowly enlarging masses without preoperative tongue weakness, they may be clinically and radiologically mistaken for primary submandibular gland tumors (5–8). Accurate recognition of the neural origin is important for surgical planning, preservation of hypoglossal nerve function, and avoidance of unnecessary removal of the submandibular gland. We report a rare extracranial hypoglossal nerve schwannoma located in the submandibular region that mimicked a pleomorphic adenoma preoperatively. This case highlights the diagnostic challenge of distinguishing neural-origin tumors from primary salivary gland lesions and emphasizes that recognition of neural origin before surgery may influence operative decision-making, potentially allowing preservation of both the hypoglossal nerve and the uninvolved submandibular gland.

## 2 Case presentation

A 44-year-old female presented to our clinic on June 19, 2024, reporting a painless left submandibular mass of three months' duration. The patient had no significant medical history, previous surgical history, or chronic medication use. There was no family history of similar tumors or hereditary tumor syndromes. No previous history of head and neck tumors or radiation exposure was reported. The patient first noticed the mass, which she described as approximately “egg-sized,” three months prior to presentation. She denied associated symptoms such as fever, erythema, swelling, localized pain, tongue numbness, or restricted tongue movement.

Upon specialist examination, an oval-shaped mass measuring approximately 4.5 cm × 5.0 cm was palpated in the left submandibular region. The mass was firm, mobile, and non-tender, and showed no significant adhesion to surrounding tissues, with no evidence of overlying skin erythema or ulceration. A comprehensive cranial nerve and oral examination was performed before surgery. The patient demonstrated normal tongue mobility without deviation, atrophy, or fasciculation. No weakness of tongue protrusion or lateral movement was observed. Other lower cranial nerve functions, including facial movement, palatal elevation, swallowing function, and shoulder elevation, were normal. Oral examination revealed no mucosal abnormalities, no floor-of-mouth swelling, and no abnormality of the submandibular duct opening. Color Doppler ultrasound revealed a hypoechoic nodule adjacent to the left submandibular gland, measuring approximately 59 mm × 36 mm × 43 mm. This lesion exhibited well-defined borders, a regular shape, and heterogeneous internal echoes with visible punctate and linear blood flow signals (Figure 1A). Contrast-enhanced computed tomography (CT) of the facial region demonstrated a slightly hypodense mass in the left submandibular region with indistinct borders from the left submandibular gland, measuring approximately 56 mm × 48 mm in maximum cross-sectional dimensions. Compression and narrowing of the left oropharynx were noted, and heterogeneous contrast enhancement was observed. No adjacent bone destruction or significantly enlarged peripheral lymph nodes were identified (Figures 1B-D). The lesion was closely associated with the submandibular gland on ultrasound and CT, leading to the initial diagnosis of pleomorphic adenoma. Retrospective assessment suggested possible compression or displacement of the gland rather than definite glandular origin; however, neural continuity with the hypoglossal nerve could not be demonstrated on available imaging. Based on these findings, a preoperative diagnosis of pleomorphic adenoma was considered.

**Figure 1:**
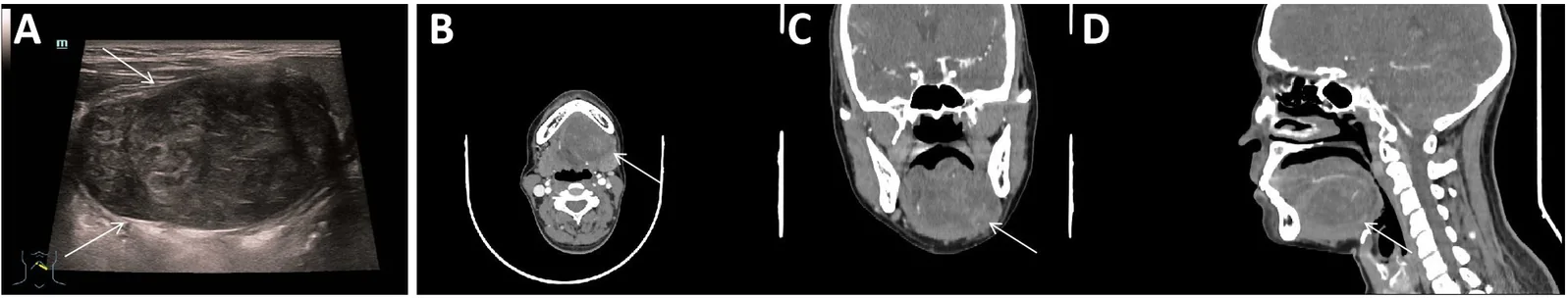
Preoperative imaging findings and retrospective assessment of the relationship between the tumor and the submandibular gland. **(A)** Color Doppler ultrasound image showing a hypoechoic nodule (white arrow) in the left submandibular region. **(B)** Axial CT image showing the left submandibular mass (white arrow). **(C)** Coronal CT image delineating the mass (white arrow) and its relation to surrounding structures. **(D)** Sagittal CT image demonstrating the extent of the mass (white arrow).

Retrospectively, the imaging findings were reviewed to reassess the relationship between the lesion and the submandibular gland. The mass appeared to be located within the submandibular space and closely contacted the gland; however, the available ultrasound and CT images did not clearly demonstrate whether the lesion originated from the glandular tissue or compressed and displaced the gland from an adjacent neural structure. The absence of definite continuity between the tumor and the hypoglossal nerve, together with the close anatomical relationship with the submandibular gland, contributed to the preoperative interpretation as a primary salivary gland tumor.

Preoperative FNAC or core-needle biopsy was considered but was not performed. FNAC was considered potentially limited because of the possibility of sampling error in a heterogeneous encapsulated lesion. Although ultrasound-guided core-needle biopsy could have provided additional diagnostic information, the potential for an inconclusive result remained. The decision for surgery was made after consideration of the imaging findings, clinical presentation, and the patient's preference. Further magnetic resonance imaging was recommended to clarify the relationship between the lesion and adjacent soft tissues; however, the patient declined because of financial concerns and reluctance to undergo additional examinations. Following completion of the preoperative assessment, the patient underwent surgical excision of the left submandibular mass under general anesthesia through a conventional submandibular approach. Because preoperative imaging suggested that the lesion was closely associated with the submandibular gland and a pleomorphic adenoma was suspected, the operation was initially planned according to a salivary gland tumor strategy. The submandibular gland was exposed and mobilized, and the submandibular duct was ligated. The lingual nerve was identified and carefully retracted superiorly. The submandibular gland was removed and submitted for histopathological examination.

After removal of the submandibular gland, the operative field was carefully inspected because the remaining lesion appeared anatomically separate from the gland. The hypoglossal nerve was subsequently identified in the submandibular space by tracing its expected anatomical course inferior to the lingual nerve and deep to the posterior aspect of the submandibular region. Exploration revealed that the tumor was attached to the inferior aspect of the hypoglossal nerve and arose eccentrically from the nerve sheath rather than involving the entire nerve trunk.

The tumor was separated from the functional nerve fascicles using meticulous capsular dissection. An extracapsular dissection plane was developed along the tumor capsule, allowing gradual mobilization of the schwannoma while avoiding direct traction, excessive manipulation, or thermal injury to the nerve. The tumor was removed by nerve-sparing enucleation from the surrounding neural structures, and the continuity of the main hypoglossal nerve trunk was preserved. No intraoperative nerve monitoring was used in this case. The tumor and the previously removed submandibular gland were submitted separately for pathological examination.

Intraoperative frozen-section examination revealed a spindle-cell neoplasm suggestive of schwannoma. Histopathological examination revealed an encapsulated spindle-cell tumor. The tumor cells were arranged in interlacing fascicles and focally showed nuclear palisading. Hypercellular Antoni A areas and hypocellular Antoni B areas were identified, with focal formation of Verocay bodies. No significant nuclear atypia, tumor necrosis, or increased mitotic activity was observed. Immunohistochemically, the tumor cells showed diffuse positivity for S-100 and SOX-10 and were negative for CD34, desmin, smooth muscle actin, epithelial membrane antigen, and cytokeratin AE1/AE3. Ki-67 staining showed a low proliferative index. These findings supported the diagnosis of schwannoma. Histopathological examination of the removed submandibular gland showed normal salivary gland tissue without evidence of neoplastic involvement. Postoperatively, tongue protrusion remained centered, without tongue weakness, atrophy, fasciculation, dysarthria, or swallowing difficulty. At the one-year follow-up, no neurological deficit or clinical evidence of local recurrence was identified (Table 1).

**Table 1:** Timeline of clinical events.

Date	Clinical event
March 2024	Patient noticed a painless left submandibular mass
June 19, 2024	Initial presentation and clinical examination
June 2024	Ultrasonography and contrast-enhanced CT performed; pleomorphic adenoma suspected
June 2024	MRI recommended but not completed; FNAC/core biopsy considered but not performed
2024	Surgical excision performed through submandibular approach
Postoperative period	Histopathology confirmed hypoglossal nerve schwannoma

## 3 Discussion

### 3.1 Literature review

To contextualize the present case, previously published reports of extracranial hypoglossal nerve schwannomas presenting in the submandibular region or adjacent upper neck were reviewed. The search included the terms “hypoglossal nerve schwannoma,” “extracranial hypoglossal schwannoma,” “submandibular schwannoma,” and “submandibular region.” In addition, Vasudevan et al. recently performed a systematic review focusing on the current diagnostic approaches and management strategies for submandibular space schwannomas, providing a broader overview of this rare entity (9).

Because the published literature on extracranial hypoglossal nerve schwannomas remains limited and heterogeneous, particularly regarding tumor origin, preoperative diagnostic evaluation, surgical approaches, and functional outcomes, the present review was intended to summarize clinically relevant features rather than provide a quantitative synthesis.

Representative reported cases of extracranial hypoglossal nerve schwannomas presenting as submandibular masses are summarized in Table 2 (10–15). Published cases have generally described painless and slowly enlarging submandibular masses; however, interpretation remains limited because of the small number of reported cases, heterogeneous clinical presentations, and inconsistent presence of preoperative hypoglossal nerve dysfunction. Preoperative misclassification as a salivary gland lesion has been reported in several cases despite the use of ultrasonography, CT, MRI, or FNAC, reflecting the substantial anatomical and radiological overlap between neurogenic tumors and salivary gland lesions in the submandibular region (16). A recent case demonstrated that ultrasound-guided core-needle biopsy can establish a preoperative diagnosis of schwannoma; however, residual hypoglossal nerve palsy occurred when part of the parent nerve was resected. In contrast, the main hypoglossal nerve trunk was preserved in the present case, and no neurological deficit was identified at one year. This comparison suggests that recognition of the neural origin before or during surgery may facilitate individualized surgical planning and potentially reduce the risk of unnecessary gland removal or nerve injury.

**Table 2:** Representative reported cases of extracranial hypoglossal nerve schwannomas presenting as submandibular masses.

Study	Age/sex	Presentation	Preoperative assessment	Treatment	Outcome/key point
Al-Mahdi et al., 2012 (13)	27/NR	Tongue and submandibular lesion	Biopsy; preoperative details incompletely reported	Combined intraoral and submandibular excision	Plexiform schwannoma; illustrates an unusual extracranial distribution
Das et al., 2021 (14)	55/F	Painless right submandibular swelling for 1 year	CT and FNAC suggested a malignant submandibular gland lesion	Submandibular gland and involved hypoglossal nerve excised	Permanent hypoglossal palsy; normal salivary gland on pathology
Parisi et al., 2023 (10)	31/F	Painless left submandibular swelling for 5 months	CT, ultrasonography, and FNAC favored pleomorphic adenoma	Transcervical excision; minor hypoglossal nerve injury	Persistent tongue deviation at 3 months
Derka et al., 2024 (9)	73/F	Asymptomatic left submandibular swelling for approximately 3 years	Ultrasonography and contrast-enhanced CT; no FNAC	Surgical excision with nerve-preserving intent	Highlights displacement of the gland and the value of recognizing a neurogenic origin
Iwai et al., 2025 (15)	52/F	Right submandibular swelling	CT, MRI, ultrasonography; core-needle biopsy diagnosed schwannoma	Intraoral excision including part of the hypoglossal nerve	No recurrence at 10 months; slight residual hypoglossal palsy
Present case	44/F	Painless left submandibular mass for 3 months	Ultrasonography and CT favored pleomorphic adenoma; MRI and biopsy not performed	Submandibular approach; gland removed; tumor dissected from the nerve with preservation of the main trunk	No recurrence or neurological deficit at 1 year

CNB, core-needle biopsy; CT, computed tomography; F, female; FNAC, fine-needle aspiration cytology; MRI, magnetic resonance imaging; NR, not reported.

Compared with previously reported cases of extracranial hypoglossal nerve schwannoma presenting as submandibular masses, the present case provides several additional clinical insights. First, the tumor was relatively large, measuring approximately 5–6 cm on preoperative imaging, yet the patient demonstrated no hypoglossal nerve dysfunction before surgery, highlighting that preserved tongue function does not exclude a substantial neural-origin tumor. Second, unlike cases in which the diagnosis of schwannoma was established before surgery, the neural origin of the tumor in this patient was recognized only intraoperatively after the lesion had been managed according to a presumed submandibular gland tumor. Third, histopathological examination confirmed that the removed submandibular gland was not involved by the tumor, emphasizing the importance of considering hypoglossal nerve schwannoma in the differential diagnosis of submandibular masses to avoid unnecessary gland resection. Therefore, this case contributes a practical lesson regarding diagnostic reasoning and surgical planning for rare extracranial hypoglossal nerve schwannomas.

### 3.2 Diagnostic challenges

Preoperative diagnosis is difficult because slowly growing extracranial lesions can expand within the cervical soft tissues without producing tongue deviation, hemiatrophy, fasciculation, dysarthria, or dysphagia. Thus, normal tongue function does not exclude hypoglossal nerve origin. In addition, a schwannoma may closely abut or displace the submandibular gland and appear to arise from the gland on ultrasonography or CT.

FNAC may be useful but can yield hypocellular or nonrepresentative material when Antoni B areas, cystic degeneration, or hemorrhage are sampled (17). Core-needle biopsy provides tissue architecture and may improve diagnostic specificity (18, 19), but its use should be individualized according to accessibility and the relationship of the lesion to major nerves and vessels. In the present case, preoperative needle biopsy was not performed because of concern regarding sampling error and a potentially nondiagnostic result. MRI was recommended to clarify the lesion's soft-tissue and neural relationships (17, 20, 21), but the patient declined because of financial concerns and reluctance to undergo additional examinations. These limitations contributed to the preoperative classification of the lesion as a pleomorphic adenoma.

From an imaging perspective, the differential diagnosis of a submandibular mass should include neural tumors, salivary gland neoplasms, lymph node lesions, vascular abnormalities, and malignant tumors. Schwannomas typically appear as well-circumscribed lesions with smooth margins and may displace adjacent structures rather than infiltrate them; however, the absence of a visible nerve continuity on routine ultrasonography or CT may lead to misinterpretation as a salivary gland tumor. Salivary gland lesions are usually considered when the mass appears closely associated with the submandibular gland, particularly when the lesion shares similar enhancement characteristics. Lymph node metastasis or other nodal diseases should be considered when irregular margins, central necrosis, multiple enlarged nodes, or invasive features are present. Vascular lesions require evaluation of enhancement patterns and vascular flow characteristics on Doppler ultrasonography or contrast-enhanced imaging. Features such as bone destruction, infiltrative growth, and extensive lymphadenopathy should raise suspicion of malignant disease. In the present case, the well-circumscribed morphology, absence of bone destruction and enlarged lymph nodes, and close relationship with the submandibular gland contributed to the preoperative diagnosis of pleomorphic adenoma, while the neural origin was only confirmed intraoperatively.

For submandibular masses with uncertain origin, preoperative diagnostic evaluation should integrate clinical findings, imaging characteristics, and tissue sampling when appropriate. FNAC is commonly used for salivary gland lesions; however, schwannomas may yield insufficient or nonrepresentative cytological material because of hypocellular Antoni B areas, cystic degeneration, or hemorrhagic changes. Ultrasound-guided core-needle biopsy may provide improved tissue architecture and diagnostic accuracy, particularly when malignancy or unusual tumor types are suspected. Nevertheless, biopsy should be individualized according to lesion location, relationship with adjacent neurovascular structures, and potential risk of injury.

In the present case, the lesion demonstrated a well-circumscribed appearance adjacent to the submandibular gland on ultrasound and CT, without obvious lymphadenopathy, bone destruction, or aggressive infiltrative features. However, imaging overlap remained between neural tumors, salivary gland neoplasms, and other submandibular space lesions. Retrospective evaluation suggested that the lesion may have displaced rather than replaced the submandibular gland; however, this distinction could not be confidently established using the available preoperative imaging. MRI with dedicated evaluation of neural continuity might have provided additional information regarding the relationship between the tumor and the hypoglossal nerve. Therefore, the absence of MRI in this case represented a limitation in preoperative anatomical characterization.

When a well-circumscribed submandibular mass appears to displace rather than replace the gland, or when intraoperative continuity with the hypoglossal nerve is identified, a neurogenic tumor should be considered. Frozen-section examination may then assist in modifying the operative strategy, but it should complement rather than replace careful anatomical assessment.

### 3.3 Histopathological diagnosis and differential diagnosis

The diagnosis of schwannoma is supported by a well-encapsulated spindle-cell tumor with alternating hypercellular Antoni A and hypocellular Antoni B areas, nuclear palisading, and Verocay body formation (Figure 3). Diffuse S-100 and SOX-10 positivity further supports Schwann-cell differentiation; the S-100 immunohistochemical staining is shown in Figure 4 (22). The principal differential diagnoses include neurofibroma, malignant peripheral nerve sheath tumor, and primary salivary gland neoplasms (23). Schwannomas generally grow eccentrically from the parent nerve and may permit separation from the functional nerve fascicles, whereas neurofibromas tend to incorporate the nerve centrally. Significant cytological atypia, necrosis, and increased mitotic activity would raise concern for malignancy; these features were not identified in the present case.

### 3.4 Surgical management and prognosis

Surgical excision is commonly considered for symptomatic, enlarging, or diagnostically uncertain extracranial hypoglossal schwannomas. However, given the benign nature and slow growth of schwannomas, conservative observation with periodic clinical and radiological follow-up may be considered in selected patients with small, asymptomatic, and radiologically benign-appearing lesions, particularly when surgical risks outweigh potential benefits. Direct visualization, meticulous dissection along the capsule, and intraoperative neurophysiological monitoring when available can reduce the risk of functional injury. If the nerve is inseparable from the tumor, the potential benefits of complete resection must be balanced against permanent tongue dysfunction, and nerve reconstruction should be considered when sacrifice is unavoidable (24).

In the present case, gland preservation was not achieved because the tumor was not recognized as a neural-origin lesion before surgery. The submandibular gland was removed based on the preoperative diagnosis of pleomorphic adenoma; only after gland removal was the separate hypoglossal nerve schwannoma identified. In the present case, the tumor arose eccentrically from the inferior aspect of the hypoglossal nerve sheath. Careful capsular dissection allowed separation of the schwannoma from functional nerve fascicles while maintaining continuity of the main hypoglossal nerve trunk (25). Figure 2B demonstrates the preserved hypoglossal nerve after tumor dissection (26). The absence of postoperative tongue deviation, weakness, dysarthria, or dysphagia at one year supports the functional value of nerve-preserving dissection. However, this case also demonstrates that preservation of the nerve alone should not represent the only surgical goal. The clinical significance of this case therefore lies not only in successful nerve preservation after tumor identification, but also in demonstrating that delayed recognition of neural origin may result in removal of an uninvolved gland. Earlier recognition of the neural origin before surgery may have allowed preservation of both the functional nerve and the uninvolved submandibular gland. Unnecessary removal of a normal submandibular gland may have potential clinical consequences, including reduced physiological salivary contribution, postoperative xerostomia, altered oral comfort, and additional surgical morbidity. Although the clinical impact may vary among individuals because of compensatory function from the remaining salivary glands, preservation of uninvolved glandular tissue remains an important consideration whenever oncologically and technically feasible. In the present case, histopathological examination confirmed that the removed submandibular gland showed no neoplastic involvement, highlighting the potential benefit of establishing the neural origin of such lesions before definitive surgery.

**Figure 2:**
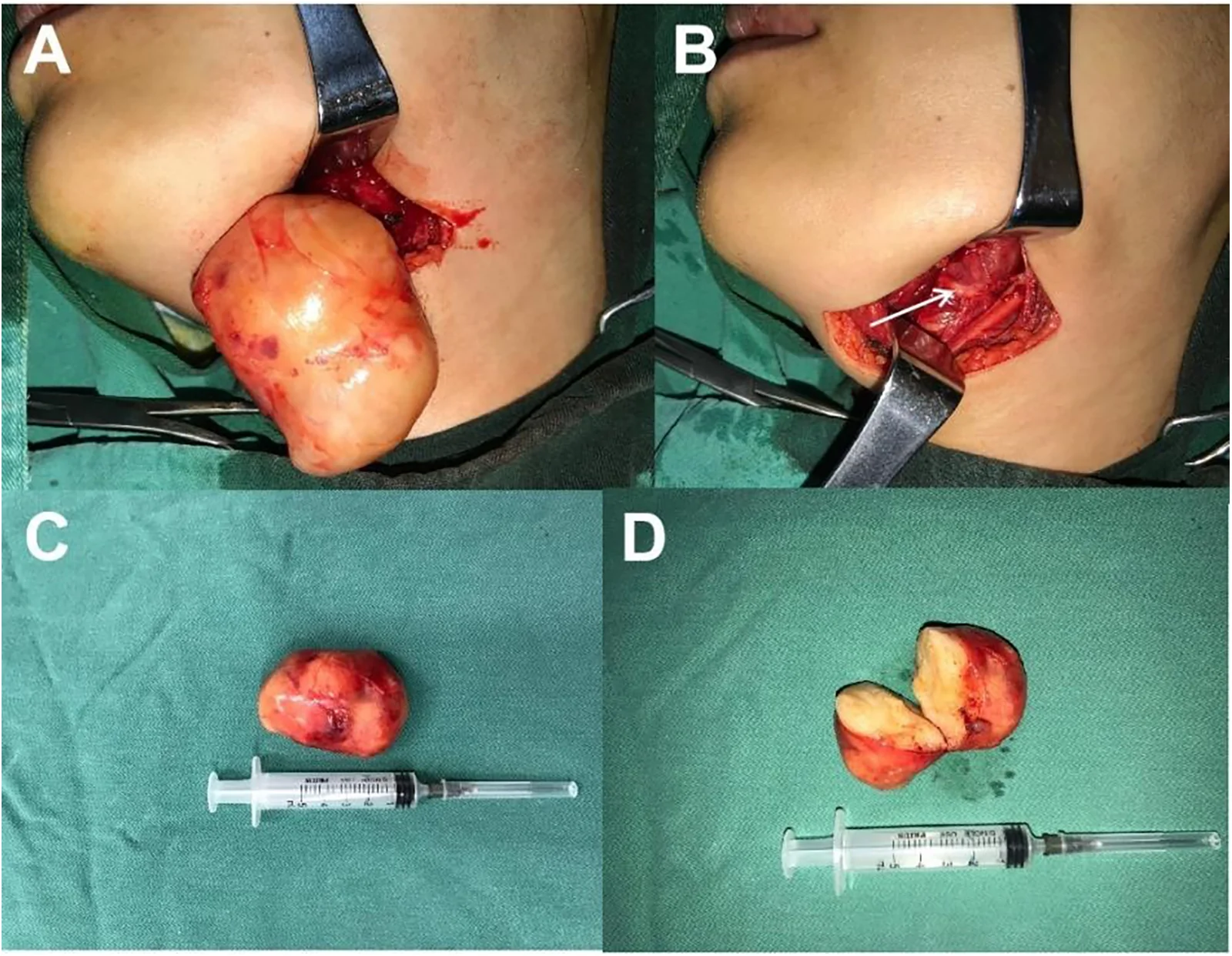
Intraoperative findings and gross appearance of the extracranial hypoglossal nerve schwannoma. **(A)** A well-encapsulated tumor exposed in the left submandibular space after removal of the presumed submandibular gland lesion. **(B)** The tumor arising from the inferior aspect of the hypoglossal nerve and being carefully dissected from the nerve sheath; the white arrow indicates the preserved hypoglossal nerve trunk. **(C)** Gross appearance of the completely resected tumor. **(D)** Cut surface of the encapsulated lesion. The main hypoglossal nerve trunk was preserved after tumor dissection.

**Figure 3:**
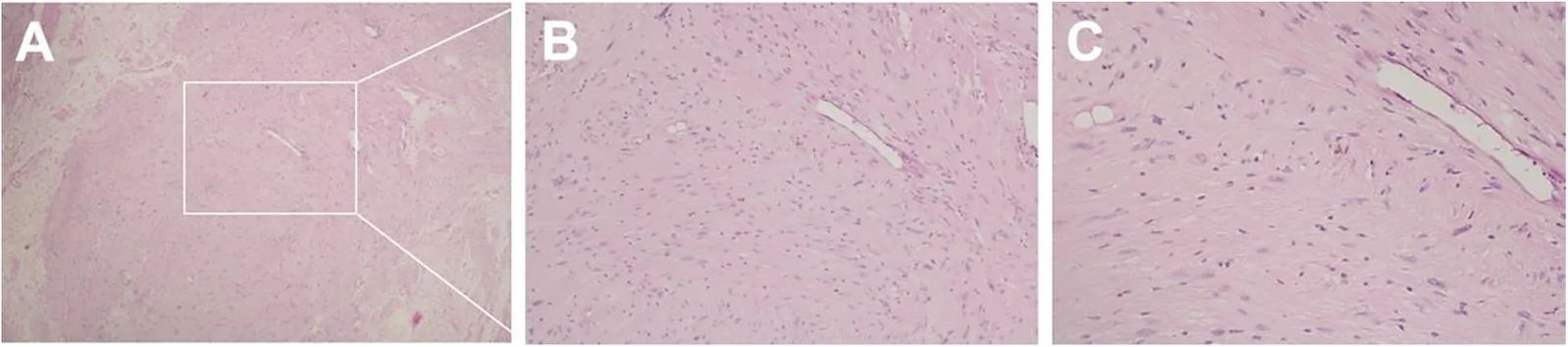
Hematoxylin and eosin (H&E) stained tissue section. **(A)** 20× Field of View; **(B)** 100× Field of View; **(C)** 200× Field of View.

**Figure 4:**
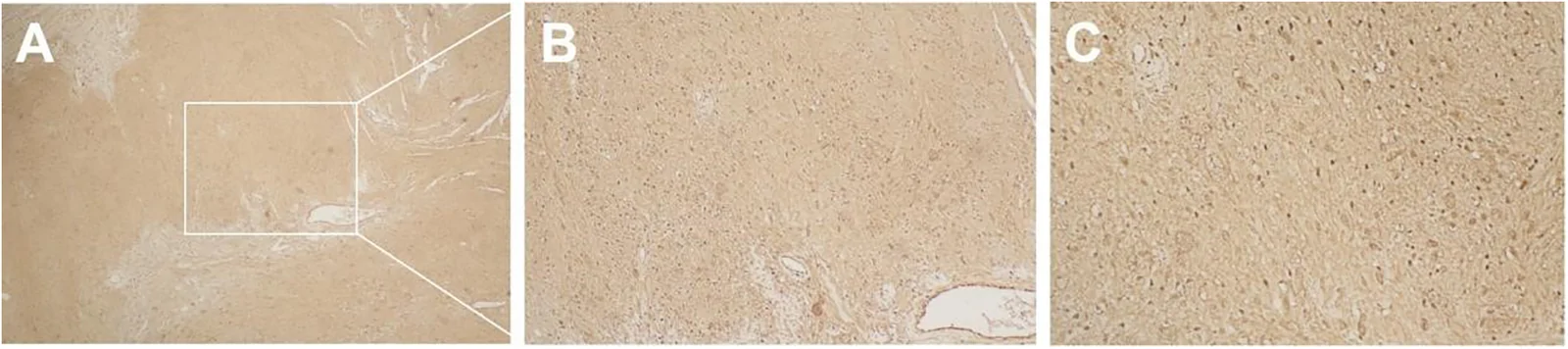
Immunohistochemical staining for S-100 in the extracranial hypoglossal nerve schwannoma. Tumor cells showed diffuse positive staining for S-100. **(A)** Original magnification, ×20; **(B)** original magnification, ×100; **(C)** original magnification, ×200.

### 3.5 Limitations

This report has several limitations. First, although the hypoglossal nerve function was clinically assessed during follow-up, objective functional assessments and validated patient-reported outcome measures were not collected, which limits the quantitative evaluation of postoperative functional recovery and quality of life. Second, the submandibular gland was removed before the neural origin of the tumor was recognized intraoperatively. Although histopathological examination confirmed the absence of neoplastic involvement of the gland, gland preservation was not achieved in this case, representing an important limitation of the current surgical course. Third, the conclusions of this report are based on a single case and a selected narrative review of previously published reports; therefore, they should be interpreted cautiously and cannot be generalized to all extracranial hypoglossal schwannomas. Fourth, MRI and preoperative needle biopsy were not completed, limiting preoperative characterization of the neural origin. Finally, follow-up was limited to one year, and longer clinical and imaging surveillance is required to better evaluate long-term recurrence risk and functional outcomes.

## 4 Conclusion

Extracranial hypoglossal nerve schwannoma should be considered in the differential diagnosis of a well-circumscribed submandibular mass, even when tongue function is normal. Previously reported cases, together with the present case, indicate that preoperative differentiation between extracranial hypoglossal nerve schwannoma and primary submandibular gland tumors remains challenging because of overlapping clinical and radiological features. MRI and appropriately selected image-guided biopsy may improve preoperative recognition, but definitive diagnosis relies on histopathological findings and demonstration of the intraoperative relationship between the tumor and the nerve. Once a neural origin is suspected, further imaging evaluation and appropriate surgical planning may facilitate preservation of both the hypoglossal nerve and the uninvolved submandibular gland. Surgical management should therefore be individualized according to tumor characteristics, symptoms, and the potential risks of intervention.

## Data Availability

The original contributions presented in the study are included in the article/Supplementary Material, further inquiries can be directed to the corresponding author.
